# KIF5C S176 Phosphorylation Regulates Microtubule Binding and Transport Efficiency in Mammalian Neurons

**DOI:** 10.3389/fncel.2016.00057

**Published:** 2016-03-15

**Authors:** Artur Padzik, Prasannakumar Deshpande, Patrik Hollos, Mariella Franker, Emmy H. Rannikko, Dawen Cai, Piotr Prus, Mats Mågård, Nina Westerlund, Kristen J. Verhey, Peter James, Casper C. Hoogenraad, Eleanor T. Coffey

**Affiliations:** ^1^Turku Centre for Biotechnology, Åbo Akademi University and University of TurkuTurku, Finland; ^2^Cell Biology, Faculty of Science, Utrecht UniversityUtrecht, Netherlands; ^3^Department of Cell and Developmental Biology, University of MichiganAnn Arbor, MI, USA; ^4^Department of Biochemistry, University of OuluOulu, Finland; ^5^Department of Immunotechnology, Lund UniversityMedicon, Lund, Sweden

**Keywords:** JNK, kinesin, axonal transport, neurons, molecular motors, phosphorylation, BDNF, SCG10

## Abstract

Increased phosphorylation of the KIF5 anterograde motor is associated with impaired axonal transport and neurodegeneration, but paradoxically also with normal transport, though the details are not fully defined. JNK phosphorylates KIF5C on S176 in the motor domain; a site that we show is phosphorylated in brain. Microtubule pelleting assays demonstrate that phosphomimetic KIF5C(1-560)^S176D^ associates weakly with microtubules compared to KIF5C(1-560)^WT^. Consistent with this, 50% of KIF5C(1-560)^S176D^ shows diffuse movement in neurons. However, the remaining 50% remains microtubule bound and displays decreased pausing and increased bidirectional movement. The same directionality switching is observed with KIF5C(1-560)^WT^ in the presence of an active JNK chimera, MKK7-JNK. Yet, in cargo trafficking assays where peroxisome cargo is bound, KIF5C(1-560)^S176D^-GFP-FRB transports normally to microtubule plus ends. We also find that JNK increases the ATP hydrolysis of KIF5C *in vitro*. These data suggest that phosphorylation of KIF5C-S176 primes the motor to either disengage entirely from microtubule tracks as previously observed in response to stress, or to display improved efficiency. The final outcome may depend on cargo load and motor ensembles.

## Introduction

Transfer of proteins to nerve terminals poses a significant long distance challenge to neurons. The classical kinesin-1 [KIF5, kinesin heavy chain (KHC), kinesin-1] motor protein comprises three subtypes (KIF5A, B, and C) (Lawrence et al., 2004) among which KIF5C is enriched in neuronal cells (Hirokawa et al., 2010). KIF5C transports cargo (mitochondria, RNA-containing granules, and vesiclular traffic) to nerve terminals at rates of ~1 μm/s (Goldstein and Yang, 2000; Vale, 2003; Hirokawa et al., 2010). A generalized increase in KIF5 phosphorylation has been associated with elevated organelle transport in axons (Hollenbeck, 1993; Lee and Hollenbeck, 1995), however the related phosphorylation sites and the underlying mechanism have been largely unresolved. KIF5 is auto-inhibited in the absence of bound cargo and while cargo bound in cells, it displays processive runs with intermittent stalling (Verhey and Hammond, 2009; Kaan et al., 2011). One can envisage that mechanisms controlling this motor will be critical for fine-tuning transport in neurons where distances from hundreds of micrometers to 1 m must be traversed and roadblocks are common (Hirokawa et al., 2010; Kapitein and Hoogenraad, 2011).

JNK regulation of axonal transport has been inferred from studies of the JIP family of JNK scaffold proteins. These scaffolds tether axonal cargo (amyloid precursor protein and ApoER2) to kinesin light chain-1 (Bowman et al., 2000; Verhey et al., 2001; Inomata et al., 2003). However, somewhat contradictory roles for JNK in regulating KIF5 transport have been described. On the one hand, following nerve ligation or excitotoxic stress, JNK is activated and anterograde traffic is inhibited, while dynein-driven retrograde transport increases (Cavalli et al., 2005), transporting stress-activated JNK to the nucleus where it phosphorylates c-Jun and initiates neuronal death (Kenney and Kocsis, 1998; Whitmarsh et al., 2001). Similarly, in the presence of pathogenic huntingtin, JNK3 inhibits anterograde transport (Morfini et al., 2009, 2013). In contrast to these studies that are carried out in stress models, a role for JNK in directing anterograde transport has also been described (Fu and Holzbaur, 2013). This study showed that phosphorylation of the JNK phosphorylation site on JIP1 leads to steric disinhibition of KIF5, and directional anterograde transport (Fu and Holzbaur, 2013). These contradictory findings indicate that JNK can both facilitate and impair KIF5 transport.

Here we examine how phosphorylation of KIF5C(1-560) by JNK alters its transport characteristics by tracking KIF5-3xmCit *in vitro* and in hippocampal neurons. We find that KIF5C is phosphorylated on S176 in the brain. The impact of this phosphorylation is to reduce the affinity of KIF5C for microtubules leading to ~50% of motors dissociating. Paradoxically however, the KIF5C(1-560)^S176D^ that remains bound shows increased processivity and speed, but also increased bidirectional movement. Yet, cargo-bound KIF5C^S176D^ shows directional plus end transport. Consistent with this, inhibition of JNK increases KIF5C(1-560) motor stalling and reduces speed while significantly increasing microtubule binding. Thus, S176 phosphorylation of KIF5C may facilitate dissociation from microtubules in the absence of bound cargo, while facilitating normal transport under conditions where cargo is bound.

## Materials and methods

### Antibodies

Polyclonal antibodies against SCG10 were previously described (Tararuk et al., 2006). Commercial antibodies were used for immunoblotting as follows: anti-kinesin heavy chain (KIF5), 1:2000 was from EMD Millipore (Cat# MAB1614 RRID:AB_94284). Anti-β-tubulin (0.1 μg/ml) was used as previously described (Westerlund et al., 2011).

### Plasmids

In every case the kinesin-1 (KIF5) sequence was derived from KIF5C obtained by PCR from mouse cDNA. For single particle tracking, pKIF5C(1-560)^WT^-3xmCit, pKIF5C(1-560)^S176A^-3xmCit, pKIF5C(1-560)^S176D^-3xmCit were prepared by insertional overlapping PCR using mutagenic and flanking primers and mouse brain cDNA, and inserted into the p3 × mCit-N1 vector as previously described (Komulainen et al., 2014). For cellular cargo assays, KIF5C(1-560)^WT^, KIF5C(1-560)^S176A^, and pKIF5C(1-560)^S176D^-3xmCit were sub cloned from 3xmCit-N1 into the β-actin-GFP-FRB vector. To obtain recombinant protein with which to assay phosphorylation and ATP hydrolysis, KIF5C(1-560) of KIF5C(1-376) was inserted into pGEX vectors using insertional PCR to generate GST-KIF5C(1-560) or GST-KIF5C(1-376). pcDNA3-HA-KLC-TPR and pcDNA3-HA-KIF5C were previously described (Cai et al., 2009; Björkblom et al., 2012). pVenus-SCG10^S62AS73A^, pVenus-SCG10^WT^ were prepared from previously described vectors (pGFP-SCG10^WT^ and pGFP-SCG10^S62AS73A^) (Tararuk et al., 2006) by insertional cloning. BDNF-Venus was a generous gift from M. Courtney, University of Eastern Finland. pEGFP-JBD was described previously (Tararuk et al., 2006).

### Protein extraction, SDS-PAGE, in-gel digestion, and phosphopeptide enrichment

A total of 150 μg of brain homogenate, or *in vitro* phosphorylated proteins, were separated on 12% Criterion gels (Bio-Rad Laboratories, Hercules, CA, USA), gels were washed in Milli-Q water, stained 1 h with GelCode (Thermo Scientific, Rockford, IL, USA), destained overnight in Milli-Q water. Each lane was manually sliced into five fractions and slices were destained then reduced and alkylated before digestion with 12.5 μg/ml sequencing grade modified porcine trypsin (Promega, Madison, WI, USA) overnight at 37°C as previously described (Björkblom et al., 2012). Peptides were eluted in 75% ACN, 1% FA. Sixty microliters of peptides were dried and immediately subjected to phospho-peptide enrichment. The peptides (ca. 50 μg/sample) were resuspended in 150 μl Binding Buffer (1 M glycolic acid, 80% ACN, 5% TFA) and mixed with 50 μl homogenous suspension of TiO2 magnetic Sepharose beads (GE Healthcare Bio-Science AB, Uppsala, Sweden) that had previously been washed 5 times in the same buffer. Peptides were equilibrated with the beads binding for 60 min at RT with gentle rocking. The beads were washed three times with 200 μl washing Buffer (80% ACN, 1% TFA) and peptides were eluted twice adding in total 100 μl 5% NH3 pH12. The pH of the solutions was lowered to <3 adding 5 μl 88% FA prior to sample clean up using C18 UltraMicroSpin columns (The Nest Group Inc., Southboro, MA, USA). Eluted peptides were then dried in a Speedvac, resuspended in 0.1% FA and then immediately analyzed by LC-MS.

### Protein phosphorylation analysis

For *in vitro* analysis, active recombinant GST-JNK3 was produced as previously described (Björkblom et al., 2012). It was used to phosphorylate GST-KIF5C(1-560) at a final concentration of 0, 0.25, 0.5, or 1.0 μM, using γ-[^32^P]ATP, as described (Tararuk et al., 2006). Phosphorylation levels were determined using phospho-imaging and kinetic plots were drawn as before (Björkblom et al., 2005).

### LC-MS/MS analysis

Peptides were separated using an Eksigent nano-LC 2D™ system coupled to an LTQ-Orbitrap XL mass spectrometer (ThermoFisher Scientific, Bremen, Germany) operated in data-dependent acquisition mode. Survey full-scan MS spectra (m/z 300–2000) were acquired in the Orbitrap in centroid mode, with a resolution of 60,000 at m/z. The spray voltage was set to 2 kV, and the temperature of the heated capillary was 180°C. After the MS1 survey scan, a multistage activation was performed by scanning for neutral losses generated by the three most intense ions. In case of neutral loss, a further stage of CID fragmentation as described above was triggered to fragment the peptide backbone. The Xcalibur software 2.0.7 (ThermoScientific) controlled HPLC, mass spectrometer and the data acquisition. All unassigned charge states and singly charged ions were rejected for fragmentation. The dynamic exclusion list was limited to a maximum of 500 masses with a maximum retention time of minutes and a relative mass window of 10 ppm.

### Data analysis

The MS/MS spectra were exported for protein identification with Mascot version 2.3 and the UniProt database (UniProt, RRID:nif-0000-00377). Cysteine carbamidomethylation was set as a fixed modification with methionine oxidation and acetylation of the protein N-terminus were added as variables. Phosphorylation of serine, tyrosine and threonine was set as variable modification when searching spectra from TiO2 enriched fractions. The FDR was set to 1% at the protein and peptide levels. The MS/MS spectra of the kinesin family phosphorylated peptides were manually interpreted to ensure correct phosphorylation site assignment.

### Cell culture and transfection

Primary cultured cortical neurons were prepared as before from newborn male and female rats (Tararuk et al., 2006; Westerlund et al., 2011). Dissociated neurons were plated in Minimal Essential Medium (MEM; Invitrogen) with 10% bovine calf serum (HyClone, Logon, US-UT), 33 mM D-glucose, 2 mM L-glutamine, 50 U/ml penicillin, and 50 μM streptomycin at the density 3000 cells/mm^2^ on poly-D-Lysine (50 μM) (Sigma-Aldrich) coated coverslips or glass-bottom dishes. Proliferation of non-neuronal cells was prevented by addition of 2.5 μM cytosine β-D-arabinofuranoside from the second day onward. Hippocampal neurons were plated in Neurobasal medium, supplemented with B27, Glutamax, 50 U/ml penicillin, and 50 μM streptomycin at the density 500 cells/mm^2^. COS7 cells were maintained in MEM supplemented with 10% (*v/v*) fetal calf serum, 2 mM glutamine, 50 U/ml penicillin, and 50 μM streptomycin. Neurons were transfected with Lipofectamine 2000 (Invitrogen) according to the manufacturer's protocol. For analysis of KIF5C variant distribution, hippocampal neurons at 4 days *in vitro* were transfected with KIF5C-3xmCit constructs as indicated, ECFP-C1 and CMV, CFP-NES-JBD, or pcDNA3-MKK7-JNK in the ratio of 4:3:3. The constructs were allowed to express for 24 h, after which cells were fixed with 4% paraformaldehyde at 4°C.

### Microtubule binding assay

The procedure was performed according to the manufacturer's instructions with modifications (Cytoskeleton, Denver, US-CO). Briefly, KIF5C(1-560)^WT^-3xmCit, KIF5C(1-560)^S176A^-3xmCit, and KIF5C(1-560)^S176D^-3xmCit were expressed in COS7 cells. Cells were lysed in PEM buffer (80 mM Pipes pH 6.8, 5 mM MgCl_2_, 2 mM EGTA, 0.15% (v/v) TX-100) containing protease inhibitors (1 μg/ml leupeptin, pepstatin, and aprotinin; 100 μg /ml PMSF) and 50 mM beta-glycerophosphate. Normalized lysates were incubated with taxol-stabilized microtubules in the presence of 2.5 mM AMP-PNP (where indicated) and incubated at RT for 30 min. Reactions were loaded onto taxol-supplemented cushion buffer and centrifuged at 100,000 × g for 40 min at RT. Soluble and insoluble fractions were lysed in *Laemmli* buffer. Equal proportions were analyzed by western blotting.

### *In vitro* single molecule total internal reflection microscopy

Cy5-labeled and unlabeled tubulin (1:10) were polymerized for 15 min, at 37°C in *P25 buffer* (25 mM PIPES, 1 mM MgCl2, 1 mM EGTA, pH 6.8) supplemented with 1 mM GTP and 4 mM MgCl_2_. After seeding, microtubules were elongated for 15 min with 10 μM Taxol (Calbiochem, San Diego, CA). KIF5C motility was carried out as described previously (Cai et al., 2007). A 30 μl flow chamber was assembled with a 22 mm slide (Thermo Fisher Scientific Inc., Waltham, US-MA) and 1 mm cover glass sealed with double-sided tape. Cy5-labeled microtubules were incubated for 5 min in the chamber and then 15 mg/ml BSA in *P25 buffer* was added for 10 min. Unbound microtubules were washed away by perfusion with 100 μl of 15 mg/ml BSA in *P25 buffer*. COS cell lysates containing KIF5C mutants prepared as previously (Cai et al., 2007) in scavenger buffer (2 mM ATP, 10 mM Glucose, 1 mM DTT, 2 mM MgCl_2_, 0.1 mg/ml glucose oxidase, 0.08 mg/ml catalase, 10 mg/ml BSA, 10 μM Taxol), were perfused through the chamber which was sealed with wax. A custom-modified microscope (Axiovert 135TV; Carl Zeiss), equipped with a 100 ×, α-plan Fluar NA 1.45 TIRFM objective, 2.5 × optovar module, CXR dichroic, and HQ540/70M emission filter (Chroma Technology, Rockingham, VT) and a back-illuminated EMCCD camera (Cascade 512B; Roper Scientific Inc., Trenton, NJ) was used. Images were acquired at 10 Hz using the 488 nm line of a tunable, single-mode, fiber-coupled Argon Ion Laser with Littrow prism (Schäfter und Kirchhoff, Melles Griot, Carlsbad, CA) at incident power of 0.55 mW. Kymographs were generated using Image J (ImageJ, RRID:nif-0000-30467). Single molecule tracking of diffraction-limited KIF5C-3mCit spots (that were clearly separated from nearby fluorescent material), was carried out only on motors that moved along stable, stationary microtubule tracks that were visualized from micrographs of Cy5-labeled tubulin (not shown), using ImageJ. Arithmetic projections were generated from KIF5C-3xmCit time-lapse acquisitions (300 frames, acquisition rate 100 ms) using Image J. These projections allow a qualitative visualization of KIF5C-3xmCit particle movement to be made. Thus, intense spots reflect low motility; feint spots reflect high motility (**Figure 2A**). Spots were tracked manually by adding the distance traveled, calculated between each acquisition until a frame was reached where no movement occurred. This distance was defined as “Run Length.” Particle speed was calculated as the distance moved [μm]/time taken [sec], as previously (Cai et al., 2007; **Figures 2B–F**). Particles that did not move at all, and particles that “quivered” i.e., very small back and forth movements without forward progression, were scored as “non-motile.” The relative expression levels of KIF5C(1-560)^WT^-3xmCit, KIF5C(1-560)^S176A^-3xmCit, and KIF5C(1-560)^S176D^-3xmCit were measured and did not differ significantly (**Figures 2G,H**).

### Inducible cargo trafficking assay

COS7 cells were cotransfected with PEX-mRFP-FKBP and KIF5C(1-560)^WT^-GFP-FRB or KIF5C(1-560)^S176A^-GFP-FRB or KIF5C(1-560)^S176D^-GFP-FRB using fugene transfection reagent and imaged after 24 h. One hundred nM rapalog was added during imaging to induce binding of FRB to FKBP domains.

### Live cell KIF5C-3mCit tracking

For time-lapse analysis (**Figure 4**), hippocampal neurons cultured in glass-bottomed dishes (WillCo Wells, NL) were transfected with LF2000 reagent at 4–5 days *in vitro* as above. To avoid tracking difficulties due to molecular clustering or high level diffusion of single KIF5C molecules, we performed imaging sessions 4 h after transfection. Neurons were imaged with a Zeiss LSM780 confocal microscope equipped with an incubator chamber (37°C, 5% CO_2_) using low laser power and half maximal pinhole size to increase the z-plane imaged. Images were acquired at 1024 × 1024 pixel resolution using a 63 × oil-immersion objective. Image acquisition was every 5 s for 10 min.

### Live cell cargo imaging in neurons

Neurons in glass-bottomed dishes (WillCo Wells, NL) were transfected with LF2000 reagent at 4 days *in vitro* and imaging was performed 24 h later. A Zeiss TIRF-3 microscope equipped with a sCMOS Orca Flash 4 camera (Hamamatsu) was used in wide-field mode to record fluorescently tagged cargos. Continuous acquisition time every 500 ms was carried out for at least 2 min for each movie.

### Motor tracking analysis

Prior to tracking, time-series were stack registered to compensate for X-Y drift and median filtered with 1 pixel radius to enhance signal to noise ratio. For directionality analysis, axons were straightened using the “*straighten*” tool from ImageJ (NIH, US). Two populations of fluorescently tagged KIF5C(1-560) molecules were observed, those with an estimated diameter of around 0.2 μm and larger clusters with an estimated diameter of 0.6 μm. Particle tracking was performed using TrackMate v2.5.4, an open-source plugin for ImageJ. For recognition of the molecules, the Laplacian of Gaussian detector was used with a 15 unit threshold for intensity level and the diameter size 0.2 or 0.6 μm with the sub-pixel localization option “on.” Only tracks where particles passed the threshold of 0.2 ± 0.02 μm and were traceable for at least five frames were chosen. For each condition, three tracks were also measured manually in order to confirm the goodness of the automatic method. Directionality was scored with the tracking of straightened axons, specifying the “X0” coordinate at the base of the growth cone. If a tracked molecule deviated (plus/minus) more than 5 μm on the X-axis for at least two sequential frames, it was scored as bi-directional. For velocity measurements, tracks with <5 μm total displacement were discarded. Pausing event ratios were calculated with the following formula: Number of 0 μm/s events/ total number of events. To estimate the proportion of diffuse movements, particles that could not be tracked frame by frame, but otherwise fitted the diameter criteria of 0.20 ± 0.02 μm and had a minimum intensity that was 15 units above background were scored.

### Immunostaining

Immunohistochemical staining of postnatal day 7 mouse brains was carried out using standard immunohistochemistry methods (Westerlund et al., 2011). SCG10 was detected using 1:1000 of an anti-SCG10 rabbit polyclonal prepared in the lab (Tararuk et al., 2006). Immunoreactivity was detected using 3, 3′-diaminobenxedine tetrahydrochloride (Sigma). Samples were counterstained using Meyer's hematoxylin (Sigma).

### ATPase measurements

For measurement of kinesin ATPase activity, GST-KIF5C(1-376)^WT^ was purified from *Rosseta 2* cells as previously described (Björkblom et al., 2012). For *step 1*, prior to ATP hydrolysis measurement, 3 μg of KIF5C(1-376)^WT^ was incubated in the presence or absence of active GST-JNK for 30 min at 30°C in 10 μl of *kinase reaction mix* (15 mM PIPES, pH 7.5, 10 mM MgCl_2_, and 1 mM ATP). For *step 2*, KIF5C(1-376) ATP hydrolysis was monitored by time-resolved absorbance. The reaction was started by addition of KIF5C(1-376)^WT^ to give a total reaction volume of 150 μl and contained 1 μg KIF5C(1-376) variant, 0.66 μM taxol-stabilized microtubules, 0.3 mM 2-amino-6-mercapto-7-methylpurine ribonucleoside (MESG), 0.15 U purine nucleoside phosphorylase (PNP), and 1 μM SP600125 (to inhibit JNK activity during the ATP hydrolysis measurements). A shift in absorbance from 330 to 360 nm was measured upon conversion of MESG to 2-amino-6-mercapto-7-methyl purine in the presence of inorganic phosphate. Absorbance measurements were made at 30 s intervals using a Multiskan Spectrum microplate spectrophotometer (Thermo Fisher Scientific).

### Statistics

Throughout, meaned data ± standard errors of the mean (S.E.M.) are shown. Significance levels were determined by Student's *t*-test or by two-way ANOVA and *post-hoc* Bonferroni test, where indicated.

## Results

### KIF5C is phosphorylated on S176 and S934 in brain

**Figure 1:**
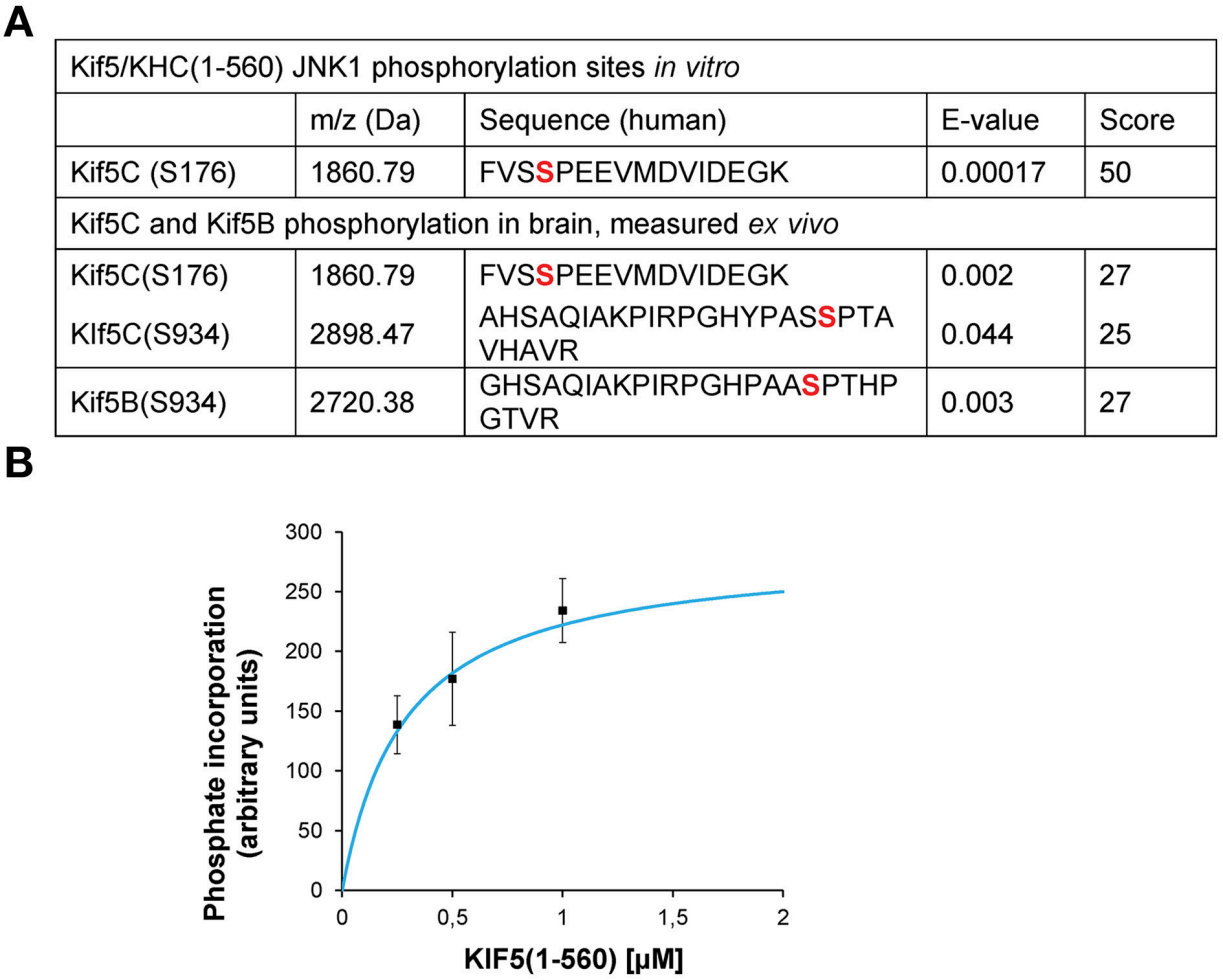
**JNK3 phosphorylates KIF5C on S176, a site that is constitutively phosphorylated in brain**. **(A)** The MS/MS phosphopeptide sequence obtained from *in vitro* phosphorylation of GST-KIF5C(1-560) by JNK3 shows that S176 (red) contained covalently bound phosphate. MS/MS analysis of phosphopeptides isolated from perinatal mouse brain showed that the same site (S176) was phosphorylated *in vivo*. Additional KIF5C and KIF5B phosphorylation sites are shown. **(B)** Kinetics of GST-KIF5C(1-560) phosphorylation by JNK3. Standard errors of the mean (S.E.M.) are shown.

To understand more about the physiological phosphorylation state of KIF5 in brain, we carried out mass spectrometry analysis. This detected covalently bound phosphate on S934 and S176 of KIF5C and S934 of KIF5B indicating that these sites are phosphorylated *in vivo* under physiological conditions (Figure 1A). We also found that JNK phosphorylated KIF5C *in vitro* on S176 (Figures 1A,B). Interestingly, while KIF5B and KIF5C both co-purified with the KHC antibody, we only detected KIF5B phosphorylation on S934, but not on S175, the site that is homologous to S176 of KIF5C (Figure 1A).

### KIF5C^S176A^ speed and run length is reduced in single particle tracking assays

**Figure 2:**
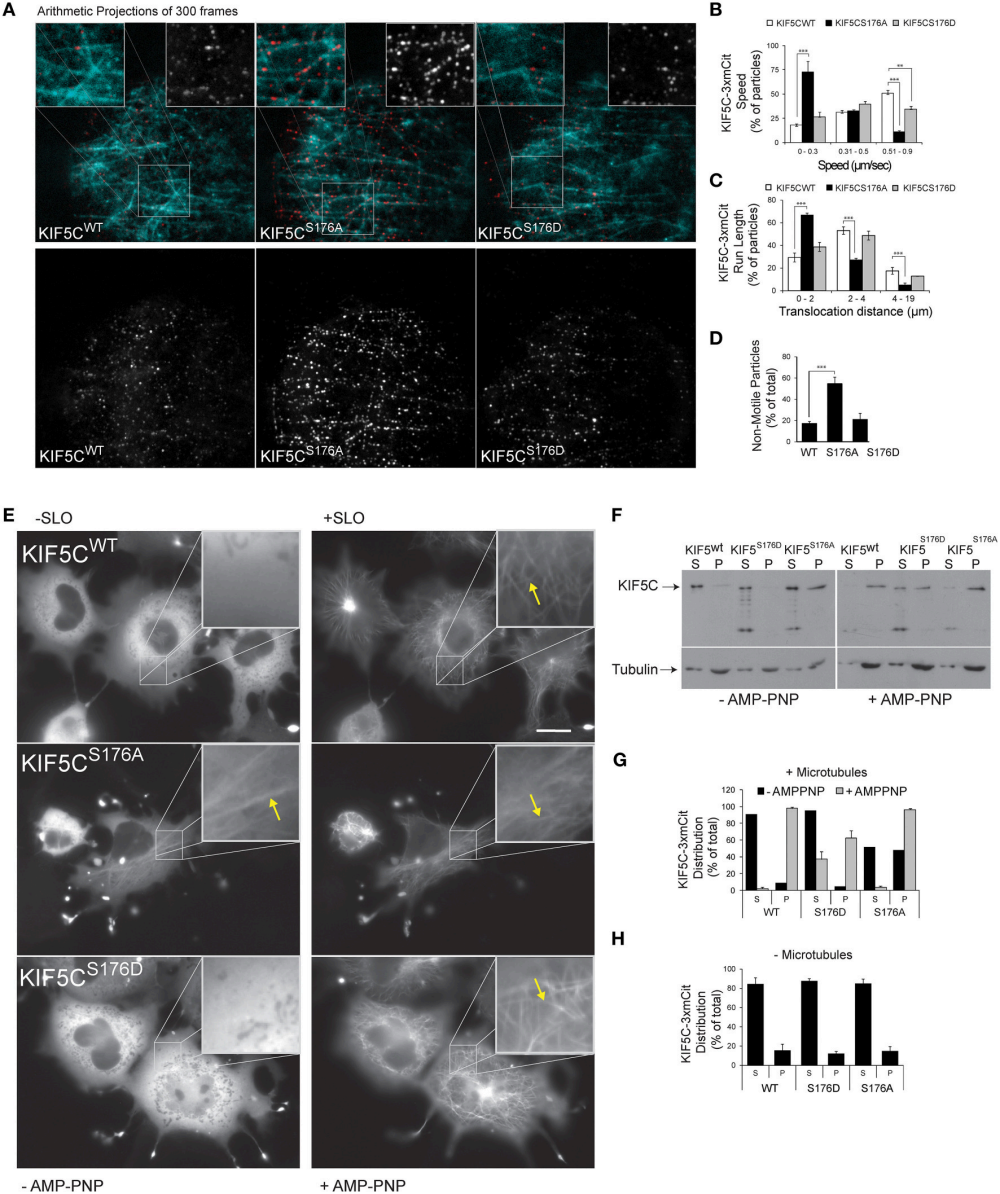
**KIF5C(1-560)^**S176iA**^ moves slower and has a shorter run length than KIF5C(1-560)^**WT**^**. To determine if S176 affected the speed of KIF5C, single particle tracking of KIF5C^WT^-3xmCit, KIF5C^S176A^-3xmCit, KIF5C^S176D^-3xmCit along taxol-polymerized microtubules was carried out. **(A)** Time series of KIF5C^WT^, KIF5C^S176A^, and KIF5C^S176D^ single particle movements was taken using TIRF microscopy. High resolution arithmetic projections from 300 frames of representative time-lapse movies are shown. Taxol polymerized microtubules are shown in cyan and kinesin particles in red (upper panels) or white (lower panels). The resulting spots do not represent kinesin aggregates, but cumulative presence of a motor at a single coordinate over time. From these projections, it is clear that KIF5C^S176A^ spots are more intense than WT or S176D, indicating longer stationary phases. **(B)** Next, KIF5C speed was measured from motors moving processively along microtubules. The distribution of speeds for KIF5C^WT^, KIF5C^S176A^, and KIF5C^S176D^ are shown. **(C)** The run length of KIF5C variants is shown. **(D)** From the same movies, the % of non-motile particles is shown for each of the KIF5C variants. KIF5C^S176A^ shows significantly increased stationary particles. For **(A–D)**, the data was extracted from time-lapse sequences carried out on three separate occasions. The number of particles counted per variant were WT = 207, S176A = 213, and S176D = 86. For **(B–D)**, mean values ± standard error of the means (S.E.M.) from five movies per variant are shown. **(E)** To visualize the effect of S176 phosphorylation on microtubule binding, COS-7 cells expressing KIF5C^WT^-3xmCit, KIF5C^S176A^-3xmCit, or KIF5C^S176D^-3xmCit were treated with or without AMP-PNP (160 μM) for 10 min. AMP-PNP-treated cells were permeabilized for 1 min using 6 nM streptolysin-O (SLO). Even without addition of AMP-PNP and SLO (left panels), KIFC^S176A^-3xmCit displayed a filamentous distribution, indicating a prominent association with microtubules (arrows). Scalebar = 10 μm. Significance was determined using Student's *t*-test. ^**^p < 0.01; ^***^p < 0.005. **(F)** Taxol polymerized microtubules were incubated with KIF5C-3xmCit variants as indicated. Microtubules and associated kinesins were pelleted by centrifugation at 100,000 × g, and fractions labeled “S” for supernatant and “P” for pellet, containing microtubule polymers. Fractions were immunoblotted with antibodies detecting KIF5 and tubulin. **(G)** Quantitative analysis from multiple experiments as described in **(F)**. This data shows that KIF5C^S176A^-3xmCit associated with the polymerized microtubule fraction (P), even in the absence of AMP-PNP. **(H)** The pelleting experiments were carried out in the absence of microtubules to determine whether the KIF5C^S176A^ variant pelleted due to aggregate formation. However, in the absence of microtubules, KIF5CS176A pelleting was equivalent to WT and S176D. Meaned data ± S.D. from two separate experiments are shown.

To determine the functional consequence of KIF5C-S176 phosphorylation, we engineered a three tandem monomeric Citrine tag (3xmCit) downstream of KIF5C(1-560), KIF5C(1-560)^S176A^, and KIF5C(1-560)^S176D^ (so as not to disturb motor-microtubule interaction), and monitored speed and run length of the motors along taxol-stabilized microtubule polymers using total internal reflection (TIRF) imaging, as previously described (Cai et al., 2007). This enables KIF5C dimers to be visualized as diffraction limited spots of around 250 nm (Cai et al., 2010). Qualitative representations of KIF5C S176 variant motility measured from time-lapse sequences of 300 frames (10 Hz acquisition frequency) are shown as arithmetic projections where each frame of the time-series is cumulatively summed (Figure 2A). Thus, more intense spots indicate longer duration of the motor at a fixed coordinate (i.e., stalling) while less intense spots indicate less time at the coordinate i.e., reduced stalling. From these projections, KIF5C^S176A^-3xmCit mutants appeared less motile, maintaining a more or less fixed x, y position compared to KIF5C^WT^-3xmCit or KIF5C^S176D^-3xmCit (Figure 2A). To directly test the motility of the S176A mutant, we measured the speed, run length and pausing of the motors (Figures 2B–D). The KIF5C^S176A^ mutant displayed lower average speed, reduced run length and increased time pausing compared to KIF5C^WT^. The majority of KIF5C^S17A^ particles (>60%) moved at speeds of between 0 and 0.3 μm s^−1^, compared to >0.5 μm s^−1^ for KIFC^WT^ (Figure 2B). Also, the % of non-motile motors was very high for the S176A mutant (Figure 2D). Notably, these assays take place under constant perfusion using TIRF imaging, and any motors that fall off of the microtubule polymers will not be detected. It is therefore striking that 50% of KIF5C^S176A^ motors remained stationary during the entire time-lapse, compared to ~20% for KIF5C^WT^ and KIF5C^S176D^ (Figure 2D). Together these data suggest that S176 is a critical residue for KIF5C function and that phosphorylation on this site may facilitate the movement of microtubule bound KIF5C.

### KIF5C^S176D^ binds poorly to microtubules whereas KIF5C^S176A^ binds strongly, even in the absence of AMP-PNP

The S176 residue of KIF5C resides within the beta5-loop8 region close to the microtubule binding domain (Amos and Hirose, 2007). This prompted us to compare the microtubule binding of the KIF5C S176 phosphorylation site mutants. We first noticed that upon over-expression in COS-7 cells, KIF5C^WT^, and KIF5C^S176D^ appeared largely diffuse in the cytosol, while KIF5C^S176A^ associated with microtubules. This was apparent even in the absence of streptolysin (SLO) permeabilization which removes the high diffuse signal due to soluble motors (Figure 2E). Following addition of the non-hydrolysable ATP analog (AMP-PNP) which traps KIF5 on microtubules (Lasek and Brady, 1985), together with SLO, all three KIF5C variants showed increased association with microtubules as expected. We next evaluated the binding of KIF5C variants to polymerized microtubules using co-sedimentation assays (Figures 2F,G). KIF5C^S176A^ pelleted strongly with microtubules even in the absence of AMP-PNP suggesting a high microtubule binding affinity. Notably, KIF5C^S176A^ did not pellet in the absence of microtubules (Figure 2H), indicating that it did not form aggregates that could lead to a false positive result in the presence of microtubules. After AMP-PNP addition, almost 100% of KIF5C^WT^ and KIF5C^S176A^ co-purified with microtubules as expected. However, only 60% of KIF5C^S176D^ co-purified with microtubules. This suggests that KIF5C^S176D^ has a lower binding affinity for microtubules. However, as the binding affinity of KIF5C^S176D^ for AMP-PNP is unknown, we cannot exclude the possibility that this is a confounding factor. Nonetheless, even in the absence of AMP-PNP, phosphorylation of S176 appears to be a critical determinant of KIF5C microtubule binding.

### Basal JNK activity is required for normal KIF5C transport to neurite tips. paradoxically however, a strongly active MKK7-JNK chimera displaces KIF5C from microtubules

**Figure 3:**
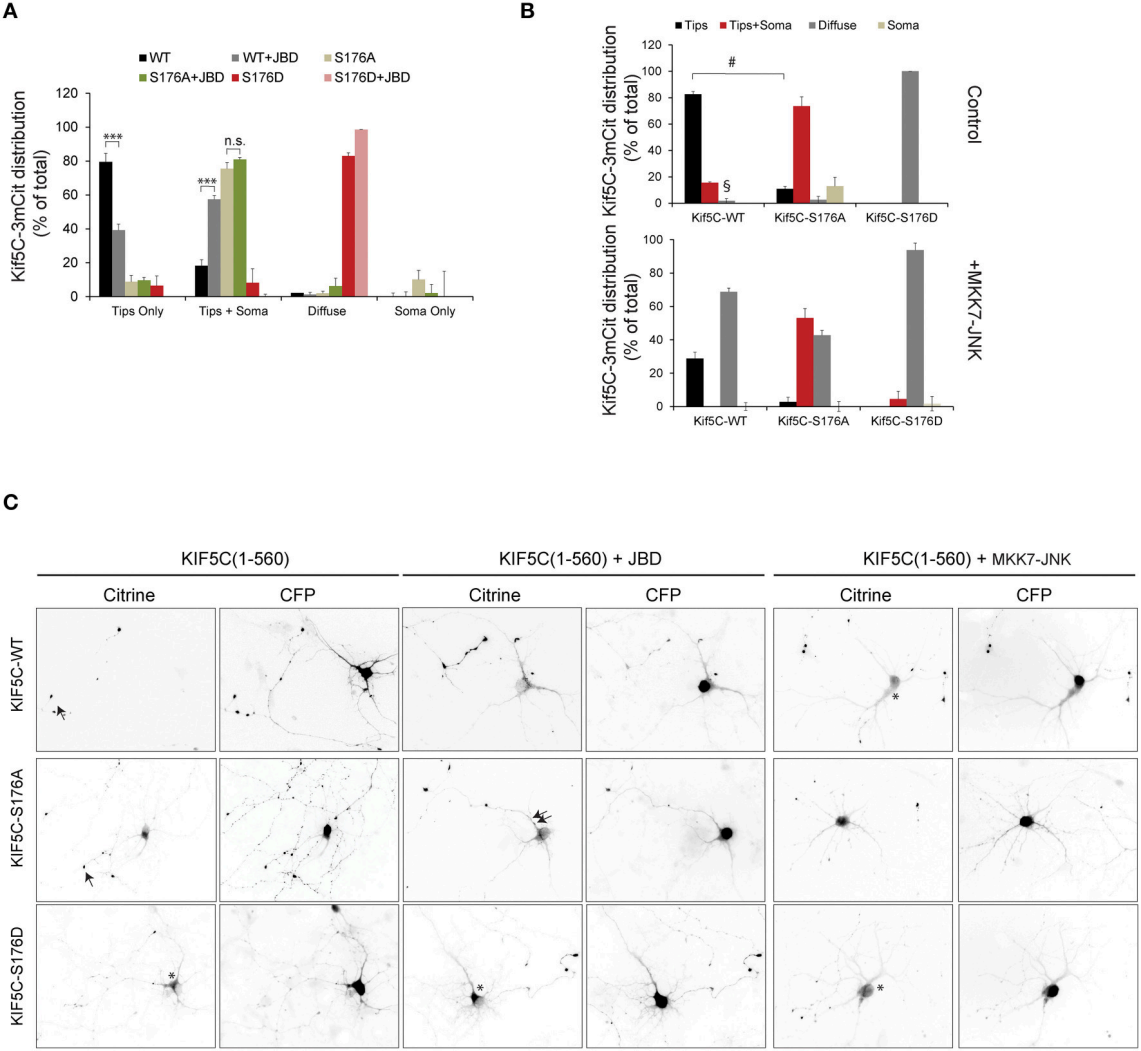
**JNK activity regulates the trafficking of KIF5C in hippocampal neurons**. **(A)** Hippocampal neurons at 4 days *in vitro* were transfected with KIF5C-3xmCit variants, as indicated and CFP (to reveal the cytosolic space). Distribution to neurite tips (tips only), soma and neurites (tips and soma), and neurites (diffuse) was measured. The influence of the JNK inhibitor JBD was also tested. Mean values ± S.E.M. from four independent experiments are shown. **(B)** Hippocampal neurons at 4 days *in vitro* were transfected with KIF5C variants as shown, in the presence or absence of the active JNK chimera, MKK7-JNK. **(C)** Representative images of citrine-tagged (KIF5C variants) and co-expressed CFP are shown. Arrow heads highlight neurite tips with enriched motors and asterisks mark diffuse motor distribution at the soma and in neurites. Mean data ± S.E.M. is shown. ^***^p < 0.005; §, *p* < 0.0001; ^#^*p* < 0.0001 between groups KIF5C^WT^ and KIF5C^WT^ +MKK7-JNK.

We next evaluated the trafficking capacity of KIF5-S176 mutants in neurons by measuring their relative enrichment in nerve tips. Hippocampal neurons were transfected with KIF5C(1-560)-3mCit variants and localization to the soma, neurites or growth cones was scored after 24 h expression (Figure 3). By this time, KIF5C^WT^ was almost entirely localized at neurite tips. KIF5C^S176A^ in contrast, localized more at the cell soma, though some reached neurite tips (Figures 3A,B). Similarly when we expressed the JNK inhibitor JBD (JNK binding domain of JIP1) in neurons, the localization of KIF5^WT^ was indistinguishable from KIF5C^S176A^ (Figure 3A). Interestingly with KIF5C^S176D^, nearly all scored neurons showed enrichment in the neurites, where KIF5C^S176D^ exhibited a diffuse signal in the cytosol (Figures 3A,C). This is consistent with its lower affinity for microtubules (Figures 2F–H). Yet KIF5C^S176D^ was also present in neuritic tips indicating that the KIF5C^S176D^ that remained bound could successfully move in an anterograde direction.

We next examined whether JNK activity affected KIF5C motility in hippocampal neurons. We therefore over-expressed an active JNK chimera (MKK7-JNK) to generate a strong and globally elevated JNK activity in the cell to mimic the stress activated kinase pool (Coffey et al., 2002). MKK7-JNK dramatically increased the proportion of diffuse motors in neurites and reduced the accumulation at neuritic tips (Figures 3B,C). These data indicate that strong activation of the kinase, as occurs in response to stress, displaces KIF5C from microtubules. This effect is largely dependent on S176 phosphorylation, as the displacement of KIF5C^S176A^ by MKK7-JNK is significantly diminished compared to KIF5C^WT^ (Figures 3B,C). The partial displacement of KIF5C(1-560)^S176A^ that is observed may be due to off-target effects of MKK7-JNK, or alternatively to phosphorylation on S934 of an endogenous KIF5C dimer partner. Nonetheless, it is clear that KIF5C^S176A^ is less susceptible to MKK7-JNK displacement than KIF5C^WT^.

### JNK activity increases KIF5C(1-560) speed and decreases stalling, while it displaces 50% of motors from microtubules

**Figure 4:**
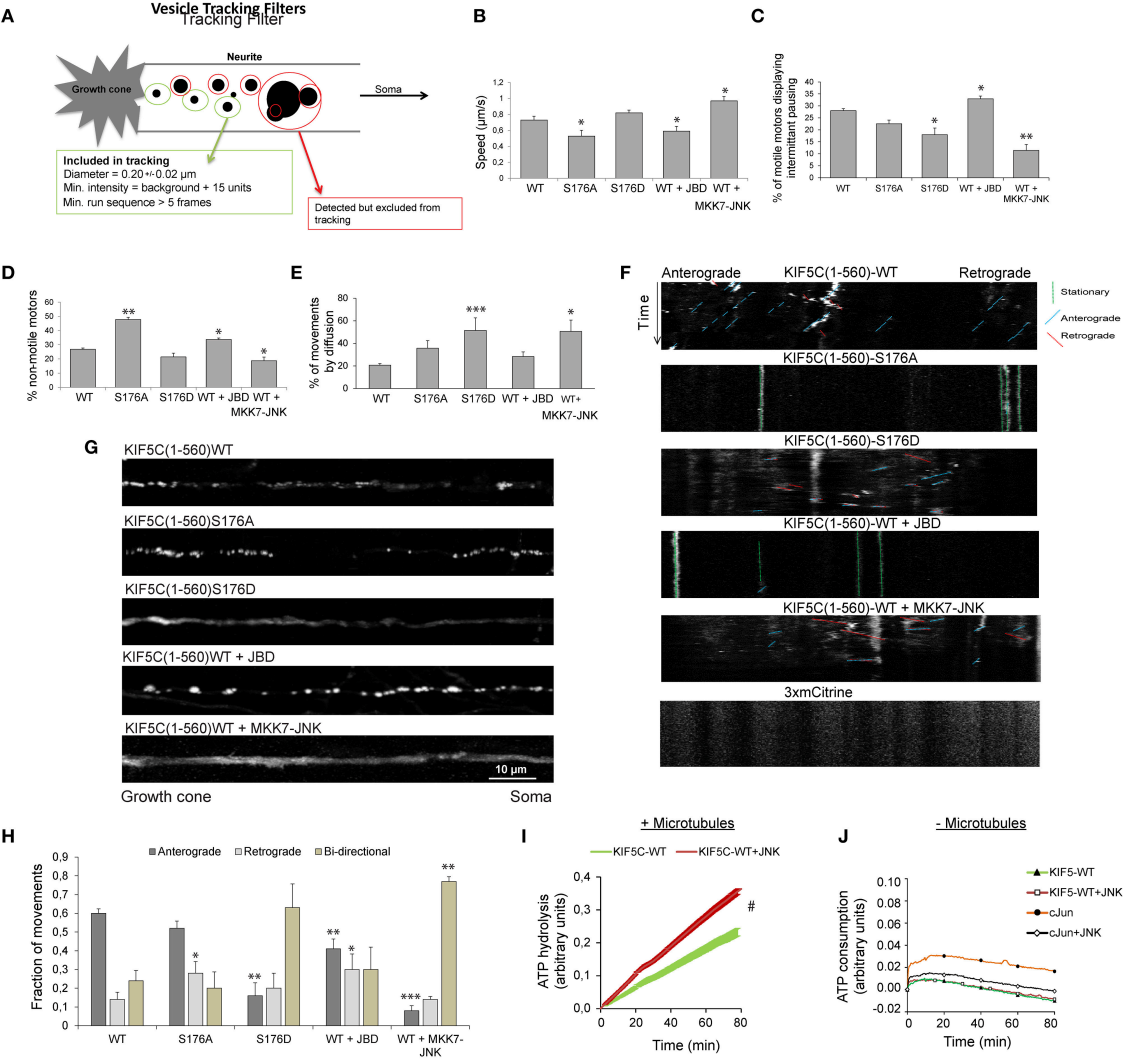
**Active JNK displaces 50% of KIF5C from microtubules while the remaining 50% displays improved motility and bidirectional movement**. **(A)** The parameters used to filter movements of KIF5C variants in 4–5 day hippocampal neurons are shown schematically. **(B)** Extrapolated motor run speeds from particle movements are plotted. **(C)** The percentage of motors exhibiting at least one non-motile event (pausing) during the 10 min acquisition is depicted. **(D)** Percentage of non-motile motors is shown. This refers to particles that fulfilled the size criteria but demonstrated <1 μm displacement during the entire recording. **(E)** The percentage of particles showing very shorts runs or diffuse movements is shown. This was defined as those particles recognized within the filter parameters for size and intensity, but lacking the minimum five connected frames **(A)**. Approximately 50% of particles in neurons expressing either KIF5C^S176D^ or KIF5C^WT^ in the presence of MKK7-JNK, displayed these characteristics. **(F)** Kymographs of KIF5C(1-560) variant movements over 10 min. **(G)** Maximum intensity projections of straightened axons from hippocampal neurons transfected with the KIF5C variants. **(H)** Directionality of motor movement, retrograde, anterograde, or bidirectional, is shown (mean data ± S.E.M.). The number of experiments (n) is indicated and the number of tracked particles is shown in parenthesis for each KIF5 variant: KIF5C^WT^ *n* = 7, (389); KIF5C^S176A^, *n* = 5 (307); KIF5C^S176D^, *n* = 6 (139); KIF5C^WT^ +JBD, *n* = 7 (235); KIF5C^WT^ +MKK7-JNK, *n* = 7 (312). **(I)** The effect of JNK on KIF5(1-376)^WT^-catalyzed ATP hydrolysis was measured in the presence of polymerized microtubules following 30 min pre-incubation with active JNK. Kinase activity was inhibited during the ATP hydrolysis reaction using 1 μM SP600125. JNK phosphorylation increased ATP hydrolysis by KIF5C^WT^. **(J)** KIF5C ATPase activity was measured in the absence of microtubules. Averaged data from three experiments is shown. Standard errors of the mean are shown for each data point. Statistical analysis was by two-way ANOVA and Bonferroni *post hoc* test. Error bars represent S.E.M.s. Significance levels, ^*^p < 0.05; ^**^p < 0.005; ^***^p < 0.0005; #*p* < 0.0001.

We next examined KIF5(1-560)^S176D^-3mCit trafficking in hippocampal neuron neurite tips using filtered tracking. A tracking filter with a maximum threshold for spot diameters of 0.2 ± 0.02 μm was used. This allowed separation of motors from large aggregates, as previously described (Lim, 1990). The filter includes only particles that are observed in more than five sequential frames (Figure 4A), thereby selecting relatively processive movements. For this purpose, we used KIF5C(1-560)3xmCit variants which encode sufficient stalk domain for dimer formation. Using this approach, KIF5C^S176A^ displayed reduced speed (Figure 4B), consistent with our earlier data in a reconstituted system (Figures 2A,B). Similarly, inhibition of JNK reduced the speed and increased the pausing of KIF5C^WT^ motors, while the active JNK chimera increased speed and reduced pausing (Figures 4B,C). Motor speed was measured from moving particles and did not take into account stationary periods. When stationary phases were measured, we found that tracked KIF5^S176A^ was twice as likely to remain immotile compared to KIF5C^WT^ (Figure 4D), again matching closely the data from the reconstituted system (Figure 2). Similarly, the JNK inhibitor increased the degree of motor stalling, while MKK7-JNK decreased it (Figure 4D).

We next estimated the proportion of tracked motors that moved by diffusion (that were excluded from Figures 4A–D). These were defined as particles with diameter of 0.2 ± 0.02 μm that could not be tracked for five sequential frames, indicating random trajectories. This subgroup was defined as particles with diameter of 0.2 ± 0.02 μm, displaying constant motility in five sequential frames. In the case of KIF5(1-560)^S176D^ and MKK7-JNK, 50% of the detected movements fitted this category, deriving either from diffuse, non-directional particle movement, or from particles with short displacements (Figure 4E). Close inspection of kymographs shows that in neurons expressing KIF5C(1-560)^S176A^ or JBD there was a clear inhibition of movement (Figure 4F). Also, KIF5C(1-560)^WT^ movements were mostly in the anterograde direction, whereas in neurons expressing KIF5C(1-560)^S176D^ or MKK7-JNK, the directionality was mixed. These differing motility patterns of KIF5C mutants can be clearly seen from maximum projection views of time-lapses (Figure 4G).

### JNK activation increases bidirectional movement of KIF5(1-560) in hippocampal neurons

As bidirectional movement is a feature of cellular transport (Hancock, 2014), we examined the directionality of KIF5C(1-560) variants. Strikingly, the majority of KIF5C^S176D^ motors showed bidirectional movement, as did wild-type motors in the presence of MKK7-JNK (Figure 4H). Bidirectional movement of motors is thought to represent a tug of war between retrograde and anterograde motors that bind the same cargo (Fu and Holzbaur, 2014). The bidirectional movements observed may represent heterodimers of KIF5C(1-560) with endogenous full length KIF5 that shares cargo with dynein motors pulling in the opposite direction. Alternatively, the bidirectional movement observed may reflect hopping of KIF5C^S176D^ to opposite polarity microtubules in the axons of these young neurons where microtubules are not yet fully polarized (Baas and Lin, 2011). Together these data indicate that in the presence of a highly active JNK (MKK7-JNK), KIF5C^WT^ moves faster, pauses less frequently and shows increased bidirectional switching (Figures 4A–H).

### JNK activity boosts KIF5C(1-376) ATP hydrolysis

In order to understand how JNK activity could increase KIF5C speed (Figure 4B), we tested whether JNK regulated ATP hydrolysis of KIF5C in a reconstituted system. For these assays, phosphate released upon hydrolysis of ATP to ADP was monitored from the coupled conversion of MESG to 2-amino-6-mercapto-7-methylpurine, inducing an absorbance shift from 330 to 360 nm. Phosphorylation of KIF5C(1-376)^WT^ by JNK significantly boosted the rate of ATP hydrolysis (Figure 4I). In these assays, KIF5C was phosphorylated by JNK (where indicated) prior to the ATPase measurements that were subsequently carried out in the presence of the JNK inhibitor SP600125. This precaution circumvented a possible false positive result from JNK ATPase activity. Indeed, ATP hydrolysis by JNK under these conditions was extremely low compared to that of KIF5C, as addition of the JNK substrate GST-cJun did not increase the absorbance at 360 nm (Figure 4J). Together these data indicate that direct phosphorylation of KIF5C by JNK augments its ATP hydrolysis rate.

### KIF5C^S176A^ displays low motility in an inducible cargo trafficking assay; KIF5C^S176D^ motility is normal

**Figure 5:**
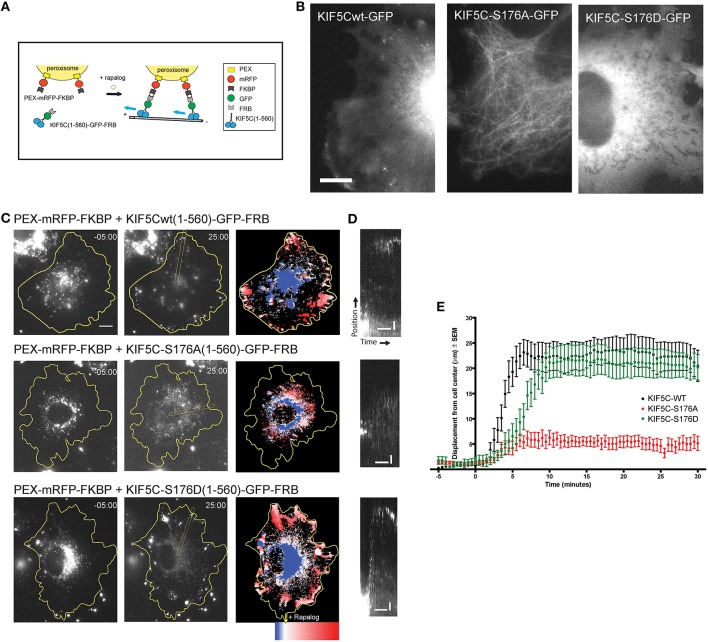
**KIF5C^**S176A**^-mediated cargo transport is reduced in living cells**. COS-7 cells were transfected with PEX-mRFP-FKBP and KIF5C^WT^-GFP-FRB or KIF5C^S176A^-GFP-FRB or KIF5C^S176D^-GFP-FRB. Time is shown in minutes. **(A)** Scheme of the inducible cargo trafficking assay. Rapalog was added at *t* = 00.00 to induce binding of KIF5C constructs to peroxisomes. **(B)** Localization of KIF5C mutants in COS-7 cells co-expressing PEX-mRFP-FKBP. Scale bar = 10 μm. **(C)** Distribution of peroxisomes before (*t* = -05:00) and after rapalog addition (*t* = 25.00). Color plots show the spatial displacement of peroxisomes colored according to time with blue frames showing peroxisome localization before rapalog, and red frames after rapalog addition. **(D)** Kymographs of cells shown in **(C)**. Kymographs were drawn from the cell center to the periphery as indicated in **(C)**. Horizontal and vertical scale bars indicate 10 min and 5 μm respectively. **(E)** Displacement graphs showing 90% of peroxisome intensity relative to the cell center over time.

To monitor transport by KIF5C in living cells, we made use of an inducible cargo trafficking assay (Kapitein et al., 2010; Figure 5A). In this assay, FRB-FKBP heterodimerization is used in combination with the cell-permeable rapamycin analog, rapalog, to trigger the binding of KIF5 motors to fluorescently labeled cellular peroxisome cargo (Figure 5A). COS-7 cells expressing PEX-mRFP-FKBP together with KIF5C(1-560)^WT^-GFP-FRB or KIF5C(1-560)^S176A^-GFP-FRB or KIF5C(1-560)^S176D^-GFP-FRB were monitored using time-lapse imaging. While KIF5C(1-560)^WT^-GFP-FRB and KIF5C(1-560)^S176D^-GFP-FRB were diffusely localized in COS-7 cells, S176?A mutation resulted in a tighter association with microtubules (Figure 5B), consistent with (Figures 2E–G). Addition of rapalog induced specific binding of the KIF5C(1-560)-GFP-FRB variants to peroxisomes at the cell center (Figure 5C). Within 2–10 min of rapalog addition, KIF5C^WT^ and KIF5C^S176D^ had delivered the peroxisomes to the cell periphery (Figures 5C,D). In striking contrast, the KIF5C^S176A^ mutant failed to displace its cargo appreciably from the cell center (Figures 5C–E). These data show that the phosphomimetic mutant displays equivalent motility of cargo-bound KIF5C in cells, whereas mutation of S176 to alanine impairs motility.

### SCG10 is a KIF5 cargo

**Figure 6:**
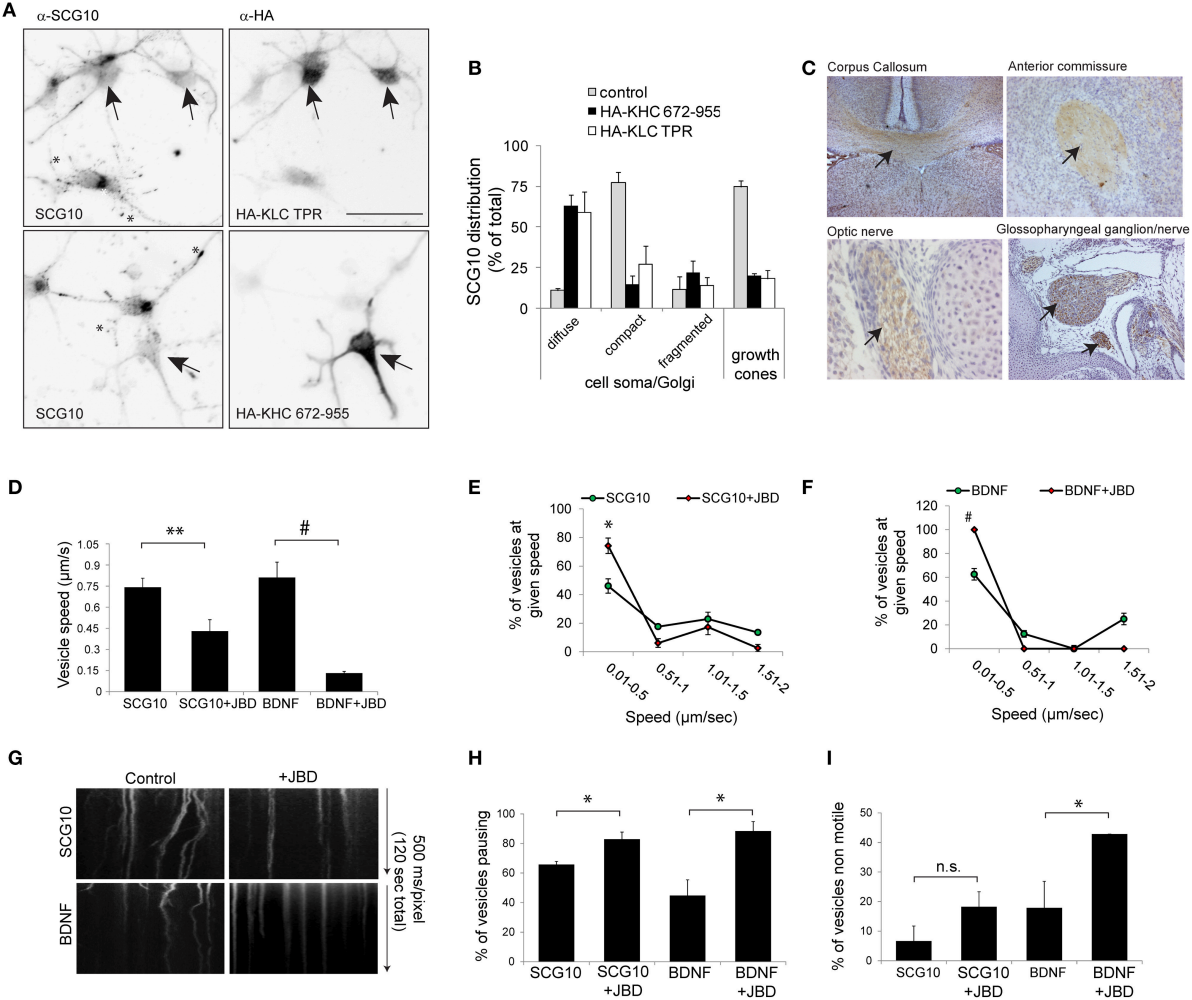
**KIF5 transport in neurons is regulated by JNK**. **(A)** To test whether SCG10 was a KIF5 cargo, hippocampal neurons were transfected with dominant negative inhibitors of KIF5 transport HA-KLC-TPR or HA-KIF5C(672-955). Endogenous SCG10 or HA-tagged dominant negative inhibitors were detected with α-SCG10 or α-HA antibodies respectively. Inverted micrographs of fluorescence composites are shown. Scalebar = 40 μm. Arrow indicates neurons that were positive for HA. **(B)** SCG10 distribution in neurons was scored at the cell soma and in growth cones. In neurons expressing dominant inhibitors of KIF5 transport, SCG10 displayed a diffuse signal in the cytosol and no enrichment at the Golgi or in growth cones (^*^). Mean values ± S.E.M. are shown from four individual experiments. Arrows point to SCG10 immunoreactivity. **(C)** Micrographs of postnatal day 7 rat brain showing enrichment of SCG10 in nerve tracts (brown). Hematoxylin was used as a counter stain (blue). **(D)** Transport of Venus-SCG10 and Venus-BDNF in cortical neurons in the presence or absence of the JNK inhibitor JBD was monitored using CCD imaging. The speed of vesicles carrying Venus-SCG10 or Venus-BDNF was averaged from multiple time-lapse movies. **(E)** Distribution plots of Venus-SCG10 transport taken from **(D)**. **(F)** Distribution plots of Venus-BDNF transport taken from **(D)**. **(G)** Kymograph plots of movements over 2 min are shown. **(H)** The % of pausing of Venus-SCG10 or Venus-BDNF is shown. Measured cargo speeds **(D–F)** do not take into account stationary vesicles. Expression of the JNK inhibitor JBD substantially increased pause frequency of both cargos. **(I)** The % of non-motile Venus-SCG10 and Venus-BDNF cargos are shown. Averaged data ± S.E.M. is shown. ^*^p < 0.05; ^**^p < 0.01; #*p* < 0.0001.

We next tested whether inhibition of JNK altered KIF5 cargo transport in mammalian neurons. We first wanted to validate whether SCG10 (superior cervical ganglion protein-10), a protein that is trafficked by fast axonal transport in peripheral neurons (Shin et al., 2012), was a bona fide KIF5 cargo. This was done using structurally distinct, dominant negative inhibitors that selectively block cargo binding to KIF5 (KHC^672−955^ and KLC^TPR^, Figures 6A,B). Expression of these inhibitors disturbed the punctate enrichment of SCG10 in growth cones, confirming that SCG10 was a cargo for KIF5 (Figures 6A,B). Consistent with this, immunohistochemical analysis showed that SCG10 was enriched in the major axonal tracts (Figure 6C).

### The JNK inhibitor JBD reduces speed and increases pausing of KIF5 cargo in neurons

We next examined the effect of JNK inhibition on KIF5 cargo transport in hippocampal neurons. Venus-SCG10 and BDNF-Venus were used as faithful reporters of KIF5 transport (Figures 6A,B; Butowt and von Bartheld, 2007). The presence of the JNK inhibitor (JNK binding domain: JBD) blocked KIF5-dependent transport in neurons (Figures 6D–G). The JNK inhibitor increased the proportion of SCG10 and BDNF cargos that stalled during a time-lapse (Figure 6H), as well as increasing the number of cargos that remained non-motile throughout (Figure 6I). Together these data indicate that constitutively active JNK facilitates KIF5 transport in neurons as exemplified by reduced speed and increased pausing of KIF5 cargo upon inhibition of JNK.

## Discussion

The KIF5 motor has attracted much attention as the prototypic kinesin that is responsible for outward (anterograde) transport in many cell types, including neurons (Vale, 2003; Hirokawa et al., 2010). Intermittent movement of KIF5 cargo is an integral feature of transport in neurons (Hollenbeck and Saxton, 2005), although the mechanisms regulating this are not fully understood. Here we demonstrate that JNK directly phosphorylates KIF5C on S176 leading to a reduced association with microtubules in cells, whereas KIF5C^S176A^ association is increased. This altered microtubule binding is reflected in the motility of the motors; thus with KIF5C^S176A^ there are more pause events, whereas S176 phosphorylation decreases pause events. These findings suggest that phosphorylation of this site provides a regulatory mechanism whereby microtubule binding, speed and stalling of KIF5C cargo can be modified in neurons.

In agreement with earlier data showing that JNK3 disrupts transport (Morfini et al., 2009, 2013), we find that S176 phosphorylation dissociates a large proportion of KIF5C motors from microtubules. However, ~50% of phosphorylated KIF5C remains microtubule bound, and displays increased run length *in vitro* and decreased pause frequency in neurons (Figures 2C, 4C). What determines the final outcome of S176 phosphorylation; microtubule dissociation or improved transport may depend on the cargo binding state. Thus, the sensitivity of a motor to phosphorylation may differ depending on the force generated. For example, motors under load, or working in populations display different motility properties (Arpağ et al., 2014). Consistent with this idea, we show that peroxisome cargo bound KIF5C(1-560)^S176D^ does not dissociate from microtubules but transports to plus end tips, though with slightly lower speed than KIF5C(1-560)^WT^ (Figure 5).

Increased retrograde JNK transport has been observed in excitotoxicity and axotomy models (Kenney and Kocsis, 1998; Whitmarsh et al., 2001; Perlson et al., 2009). This is understood to enable stress activated JNK to elicit a transcriptional stress response in the nucleus. In our experiments we observe increased bidirectional movement of KIF5C(1-560)^WT^ following JNK activation. Assuming that exogenous KIF5C forms dimers with endogenous full length, cargo-bound kinesin-1, this bidirectional movement can be the consequence of a “tug of war” between dynein motors sharing the same cargo (Fu and Holzbaur, 2014). It may be that under stressful conditions, the lowered affinity of S176 phosphorylated KIF5C increases the probability for retrograde movement, dependent on the population of motors bound. Additionally, this switch to bidirectional movement is reminiscent of the increased minus end motility displayed by KIF5B when phosphomimetic on S175 (Deberg et al., 2013), and is consistent with the “priming” function that we propose for JNK phosphorylation of the motor domain.

We show here that JNK activity facilitates transport of KIF5 cargos SCG10 and BDNF in neurons. JNK1 has previously been implicated in facilitation of transport through phosphorylation of the KIF5 adaptor JIP1. This leads to steric disinhibition of KIF5 (Fu and Holzbaur, 2013). The two events, adaptor and motor domain phosphorylation, may be coordinated to ensure optimal transport under certain cargo-bound conditions. Here we show that JNK3 phosphorylates KIF5C with high efficiency *in vitro*. It is entirely possible that JNK1 and JNK3 cooperate to facilitate transport via two distinct mechanisms, steric disinhibition and motor domain regulation respectively. Regardless of which mechanism is involved, an important conclusion that can be derived from this study is that in mammalian neurons, physiologically active JNK, which is present in the cytosol of neurons (Coffey et al., 2000; Cavalli et al., 2005; Oliva et al., 2006), is required for KIF5C dependent transport of BDNF, a critical regulator of synaptic plasticity and mood (Duman and Monteggia, 2006; Figures 6D–I).

The three subtypes of KIF5, A, B, and C share similar though not identical sequences with overall homology in the motor domain ranging from 30 to 60%. The “S176-P177” motif is evolutionarily conserved among the KIF5 subtypes from squid (*Loligo*) to human (Morfini et al., 2009). One could imagine therefore that KIF5A, B, and C would behave similarly upon phosphorylation of this residue. Consistent with our findings with KIF5C in neurons, phosphomimetic KIF5B^S175^ alters directionality *in vitro* (Deberg et al., 2013). Yet this study also demonstrates decreased velocity of phosphomimetic KIF5B^S175^ *in vitro*, whereas in neurons we observe improved overall speed of KIF5C(1-560) with a phosphomimetic variant or upon JNK activation. Again, this may be context dependent and the cargo load and/or motor ensemble associated with KIF5C in neurons may define the motor's response to phosphorylation on this residue. Also, it is possible that in spite of overall sequence similarity between KIF5B and C, they serve non-overlapping functions in mammals (Tanaka et al., 1998; Kanai et al., 2000). This may reflect different sensitivity to regulators.

**Scheme 1:**
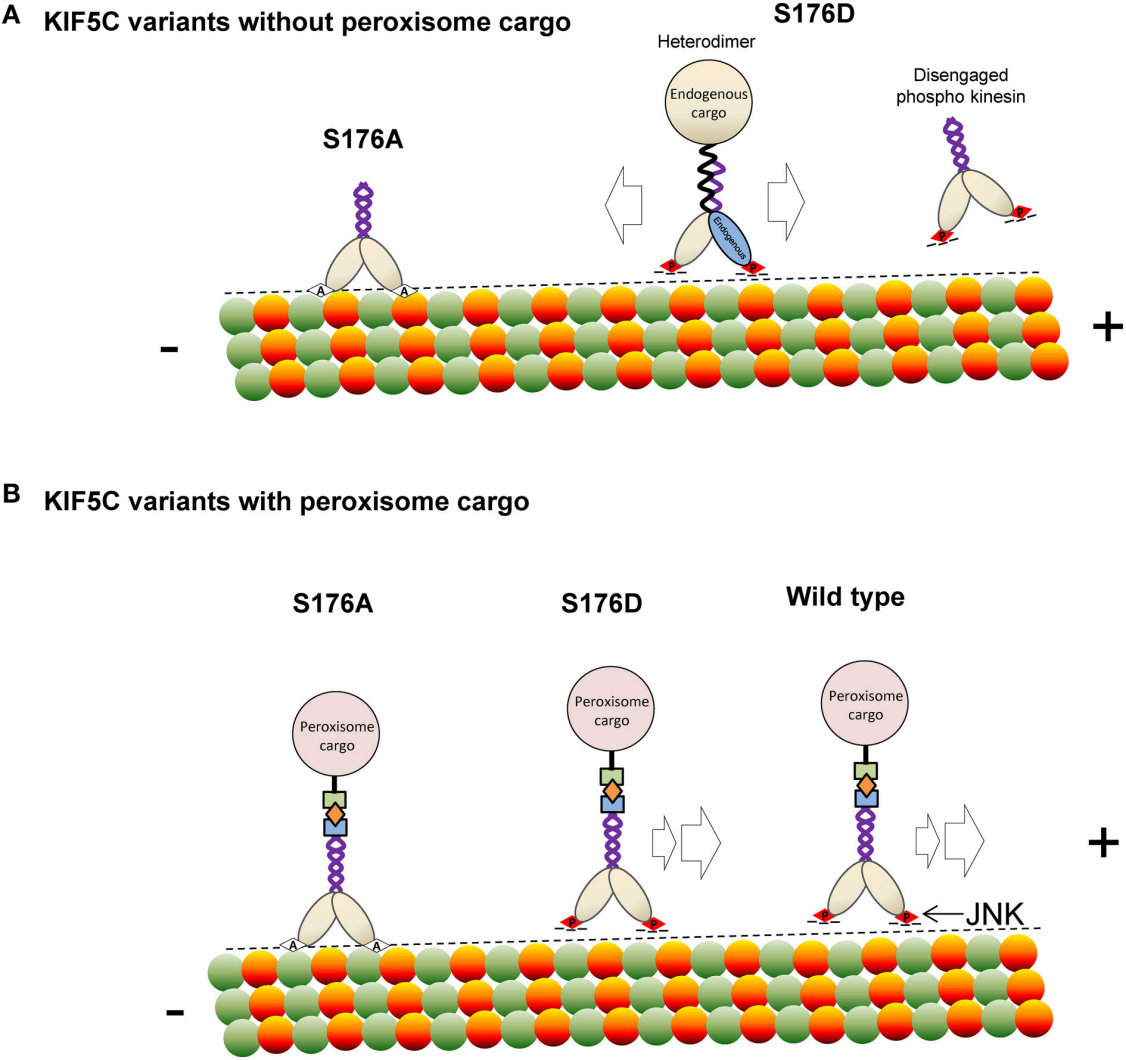
**A model depicting how phosphorylation of KIF5C on S176 may regulate microtubule binding and motility**. **(A)** In the absence of rapalog-tethered cargo, S176 phosphorylation causes either complete disengagement of KIF5C(1-560) from microtubules or increased bidirectional shuffling. In contrast, non-phosphorylated KIF5C(1-560) associates tightly with microtubules. **(B)** In the peroxisome cargo-bound state, S176 phosphorylated KIF5C(1-560) transports to microtubule plus ends, whereas dephosphorylated KIF5C(1-560) is bound tightly to microtubules resulting in an immobile state. As a consequence, phosphorylation of S176 can facilitate plus-end cargo transport by KIF5C(1-560).

In summary, we report that JNK phosphorylates KIF5C(1-560) leading to reduced microtubule binding. This modification can displace the motor under conditions of stress, such as those imposed by hyper activation of JNK, or when cargo is not bound (Scheme 1). At the same time, S176 phosphorylation facilitates efficient movement and directionality of the motor in the presence of bound cargo. This phosphorylation event is important as mutation of S176 of KIF5C to alanine, or inhibition of endogenous JNK, stalls the motor (Scheme 1). Our findings indicate that JNK is an important regulator of KIF5C dependent transport in neurons.

## Author contributions

All authors listed, have made substantial, direct and intellectual contribution to the work, and approved it for publication.

### Conflict of interest statement

The authors declare that the research was conducted in the absence of any commercial or financial relationships that could be construed as a potential conflict of interest.
